# Mechanical failure of a femoral lengthening nail: a case report

**DOI:** 10.1186/s13037-018-0154-4

**Published:** 2018-05-03

**Authors:** WeiWei Wu, Kevin M. Kuhn

**Affiliations:** 34800 Bob Wilson Drive, San Diego, CA 92134 USA

**Keywords:** Femoral lengthening nail, Distraction osteogenesis, Nonunion

## Abstract

**Background:**

This case report describes the mechanical failure of a femoral lengthening nail in order to allow early recognition and prevent its occurrence.

**Case presentation:**

A femoral lengthening nail was used to achieve goal distraction length over eight weeks and sustained a mechanical failure during progressive weightbearing. Re-distraction was attempted with subsequent non-weight-bearing status. Although the patient was not able to attain the original goal length, radiographs demonstrated stable shortening of the patient’s right lower extremity along with early signs of incomplete regenerate bone. She eventually received exchange nailing with bone graft.

**Conclusion:**

Post-operative protocol and weight-bearing status should be further researched and standardized after re-evaluating the mechanical stiffness of the intramedullary nail construct.

## Background

Congenital and acquired leg length discrepancy have often been treated by distraction osteogenesis, traditionally with external fixation frames. Recently, Intramedullary Nail (IMN) lengthening devices have been developed to allow limb lengthening with the advantages of intramedullary fixation while avoiding the known complications of external fixation (i.e. pin tract infections and patient acceptance). There have been few studies analyzing the outcomes of these devices [1–6]. This report describes a case of post-lengthening mechanical failure of the PRECICE® nail (Ellipse Technologies, Inc., Irvine, CA, USA). To the author’s knowledge, this complication has not been reported previously, and is described herein to help allow early recognition and prevent its occurrence.

## Case presentation

**Fig. 1:**
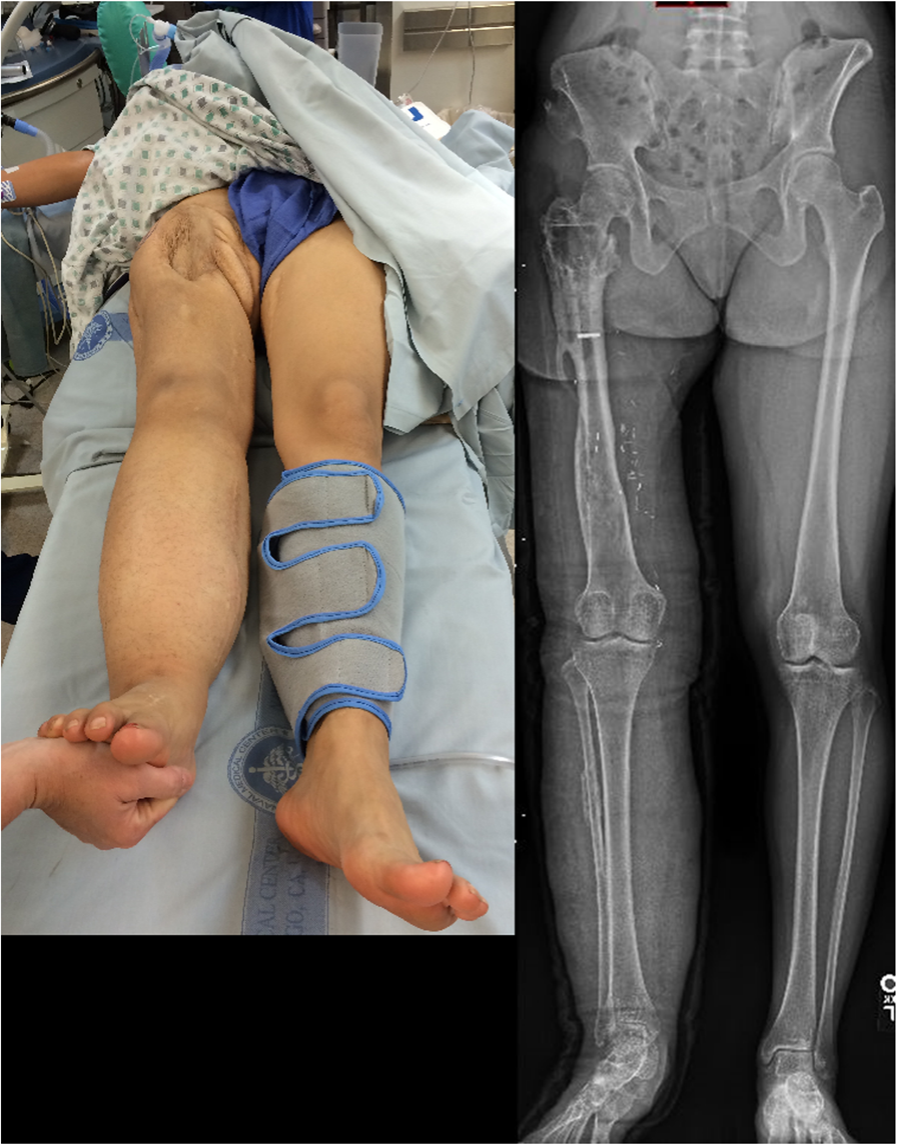
Initial clinical presentation and long leg alignment films demonstrating a 3 cm leg-length discrepancy

**Fig. 2:**
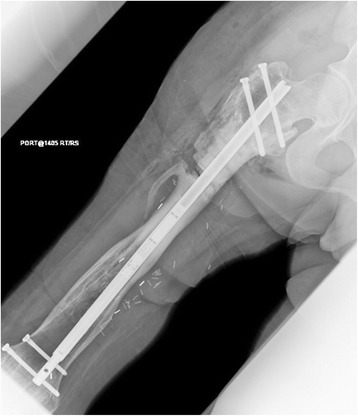
Post-operative portable films of right femur with PRECICE® nail

A 24 year-old female was struck by a trolley as an adolescent and sustained a severe Grade 3C open femur fracture. After a multistage limb salvage procedure which required vascular repair and soft tissue reconstruction, she presented to our clinic more than a decade after the injury. The patient stood 1.6 m and weighed 64.8 kg, with a calculated Body Mass Index (BMI) of 25.3. She acquired a leg length discrepancy of 30 mm, right shorter than left, had right hip pain, walked with a limp and complained of symptomatic hardware about her hip (Fig. 1). She underwent a proximal femoral osteotomy and placement of a 10.7 mm antegrade PRECICE® expandable femoral IMN (Fig. 2). Although she required four units of pRBC transfusion post-operatively, she tolerated the procedure well with no other complications. The femur was left in situ after the osteotomy to allow surgical wounds to heal.

**Fig. 3:**
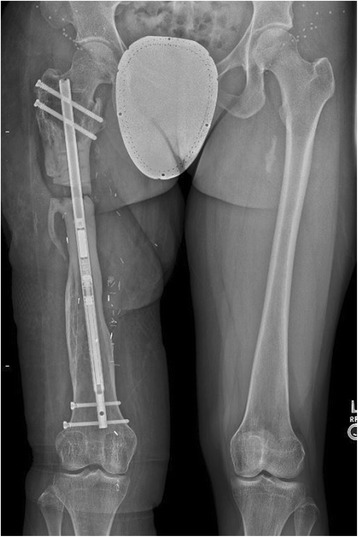
Long leg weight-bearing plain films demonstrating femur after one week of lengthening

**Fig. 4:**
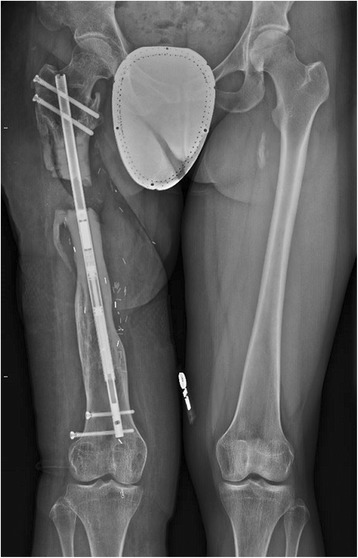
Long leg weight-bearing plain films demonstrating femur after four weeks of lengthening

Ten days post-operatively, she followed up with uncomplicated healing wounds to begin her lengthening, 1 mm per day for a goal of 30 mm total lengthening. She was followed weekly with physical exams and plain film radiographs (Fig. 3). After four weeks of lengthening, physical exam demonstrated equal iliac crest height on standing and symmetric rotation. Bilateral long leg Weight-Bearing (WB) plain films demonstrated that the right femur was within 2 mm of length compared to the left femur (Fig. 4) and regenerate was visible in the osteotomy gap. There was some backing out of one of the distal interlocking screws. We believed that she had achieved goal length and started her on 25% WB and physical therapy for hip and knee range of motion and strengthening.

**Fig. 5:**
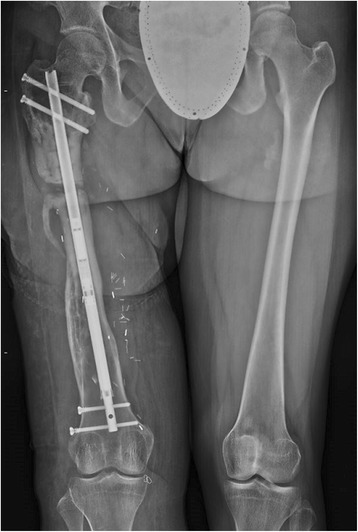
Long leg weight-bearing plain films demonstrating femur at eight weeks after lengthening with mechanical shortening of nail

**Fig. 6:**
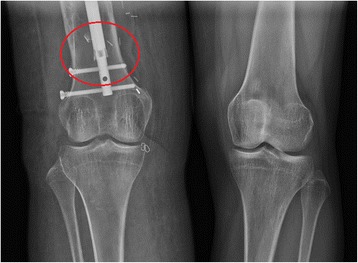
Enhanced view of distal nail, with a red circle demonstrating flaring of the nail flange that was not present in previous films

Four weeks afterwards, the patient reported increased pain in her right thigh and felt that her right leg had shortened. On physical exam, she was grossly 2 cm short on her right lower extremity with tenderness to palpation over the distal interlocking screw. Plain films demonstrated a 25 mm leg length discrepancy (Fig. 5). Closer inspection of the radiographs revealed that the flange of the nail was flared distally (Fig. 6) and the lengthening mechanism had failed.

**Fig. 7:**
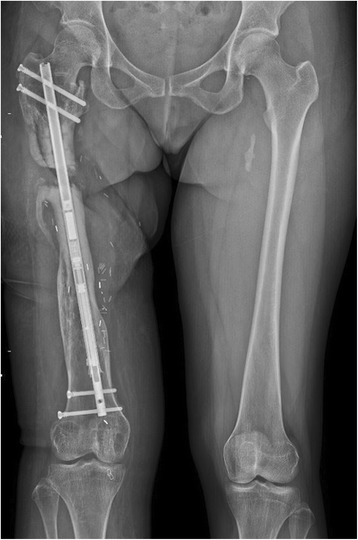
Long leg weight-bearing plain films demonstrating femur at ten weeks after restarting lengthening

**Fig. 8:**
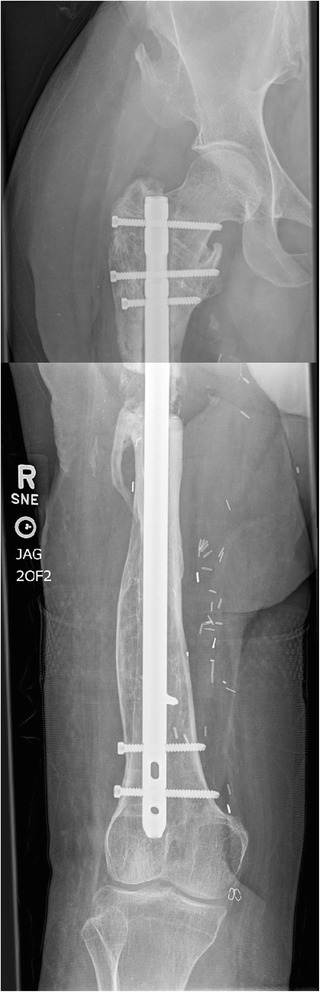
Final construct after exchange nailing. Most recent films from January 2016

We attempted to resume her original lengthening protocol, with the hope of regaining some of the lost length. She continued the protocol and stayed non-WB for the duration of her lengthening, however only an additional 5 mm were gained (Fig. 7) as the mechanism would not lengthen any further, presumably due to the mechanical failure. Over the next several months, she continued to have pain in her right thigh and was unable to weightbear without crutches. Her osteotomy went on to nonunion and she eventually underwent exchange nailing (Fig. 8).

## Discussion

Congenital and acquired leg length discrepancy have often been successfully treated with distraction osteogenesis at a rate of 1 mm/day according to Ilizarov’s principles [7, 8]. Traditionally, external fixation frames have been a reliable and accurate tool, however are cumbersome devices that need to be worn for several months and are associated with a variety of complications. These problems include pin tract infections, hardware breakage and loosening, soft tissue tethering, scarring, and joint stiffness [9].

During the last couple of decades, intramedullary telescopic nails insertion after osteotomies have gained popularity. However they are also not without their complications. Albizzia® (Medinov-AMP, Roanne, France) is a spring-and-ratchet assembly that requires 15° of rotation for incremental distraction. Problems included anesthesia required during ratcheting of nail, mechanical failure requiring nail exchanges, bone grafts needed, and infection [2, 3]. The Intramedullary Skeletal Kinetic Distractor (ISKD; Orthofix, Inc., Lewisville, TX, USA) is an FDA-approved roller-clutch-threaded-rod design that requires 3-7° of rotation for distraction. However, when it was compared with the hybrid technique of external fixation and lengthening over a nail, ISKD produced poorer outcomes as it had an unreliable control of distraction, leading to increased final leg length discrepancy [4, 6]. ISKD also has the highest rate of mechanical failure amongst IM lengthening nails with fracture of the device and/or its external parts [5]. Fitbone® (Wittenstein, Igersheim, Germany) employs an electric motor mechanism, which is only activated when an external transducer is placed directly over a subcutaneous antenna/receiver. This allows surgeons strict control over the rate and rhythm of distraction by programming the transducer’s number and duration of radiofrequency activations. Accurate distraction control is critical as too rapid a process can lead to pain with distraction, nonunion, nerve injuries, and joint contractures, while too slow a process risks premature consolidation [10].

The PRECICE® nail (Ellipse Technologies, Inc., Irvine, CA, USA) was introduced in 2011 with a novel magnetic actuator drive design. It is similar to the Fitbone® in that the surgeon can control the distraction by programming the handheld external activator. However, it has the additional advantages of being approved by the FDA, able to lengthen or shorten, and does not require a subcutaneously imbedded receiver or cable. Early studies in evaluating the outcomes reported high rates of distraction accuracy, moderate temporary loss of joint range of motion that was gained back by physical therapy, and low rate of nonfunctional distraction mechanism that was treated with a nail exchange [1].

According to Ilizarov’s principles, postoperative management with patients is limited to touch-down weight bearing immediately after surgery. One of the main advantages of lengthening over an intramedullary nail is to allow early WB as the nail is a load sharing device [11]. Femoral distraction begins approximately five to ten days postoperatively at a rate of 0.33 mm three times daily or 0.25 mm four times daily. Patients follow up in clinic every one to two weeks during the distraction phase and every four weeks during the consolidation phase. Full weight bearing is allowed when corticalization of three of four cortices is seen on biplanar radiography. Typically, the telescopic implant is removed one year after surgery, provided there is solid circumferential healing of the regenerated bone.

Our unique case of hardware failure post-distraction contributes to the mechanical complications and post-operative management protocol facing the PRECICE® nail system. This complication resulted in added expense to the medical system, likely may have contributed to the nonunion of the osteotomy and need for secondary surgery, and not to mention the added physical and emotional stress to the patient. To our knowledge, there are currently no studies detailing post-operative WB status and rehabilitation following PRECICE® nail insertion or evaluating the mechanical properties of the nail during loading [12]. This failure likely demonstrates that the device may not be strong enough to withstand even partial weight bearing in a non-obese patient during the consolidation phase, which has been touted as one of the primary benefits of lengthening with an intramedullary device. For future cases, in order to avoid this problem, consideration may be given to over-reaming to allow placement of a larger PRECICE® nail, augmenting fixation with a submuscular plate once desired length has been achieved, or exchanging the nail for a standard locked IMN once the lengthening protocol is complete.

## Conclusion

Post-operative protocol and weight-bearing status should be further researched and standardized after re-evaluating the mechanical stiffness of the intramedullary lengthening nail construct.

## Abbreviations

BMI: Body mass index
IMN: Intramedullary nail
ISKD: Intramedullary Skeletal Kinetic Distractor
WB: Weight-bearing
